# *Mycobacterium tuberculosis* and the host cell inflammasome: a complex relationship

**DOI:** 10.3389/fcimb.2013.00062

**Published:** 2013-10-09

**Authors:** Volker Briken, Sarah E. Ahlbrand, Swati Shah

**Affiliations:** Department of Cell Biology and Molecular Genetics, University of MarylandCollege Park, MD, USA

**Keywords:** *Mycobacterium tuberculosis*, ESX-1, IFN-β, inflammasome, IL-1β, NLRP3, AIM2

## Abstract

The production of IL-1β during the infection with *Mycobacterium tuberculosis* (Mtb) is important for successful host immune defense. In macrophages and dendritic cells the host cell inflammasome is crucial for generation of secreted IL-1β in response to Mtb infections. In these cell types Mtb infection only activates the NLRP3-inflammasome. New reports demonstrate that nitric oxide has an important function in the negative regulation of the NLRP3-inflammasome to reduce tissue damage during Mtb infections. The type I interferon, IFN-β, is induced after Mtb infections and can also suppress NLRP3-inflammasome activation. In contrast, IFN-β increases activity of the AIM2-inflammasome after infection with intracellular pathogens such as *Francisella tularensis* and *Listeria monocytogenes*. Recent results demonstrate that non-tuberculous mycobacteria but not virulent Mtb induce the AIM2-inflammasome in an IFN-β dependent matter. Indeed, Mtb inhibits AIM2-inflammasome activation via its ESX-1 secretion system. This novel immune evasion mechanism may help Mtb to allow the induction of low levels of IFN-β to suppress the NLRP3-inflammasome without activating the AIM2-inflammasome.

## Introduction

IL-1β is important for host immune defense against Mtb, since several studies demonstrated that IL-1β and IL-1-receptor knock-out mice are more susceptible to Mtb infections (Mayer-Barber et al., 2010, 2011; Mcelvania Tekippe et al., 2010). According to some studies IL-18 has no or only a minor role in host defense against Mtb but more recently one report showed an increased susceptibility of IL-18 but not IL-18R knockout mice [for review (Cooper et al., 2011)]. In macrophages and dendritic cells the production of mature IL-1β and IL-18 is dependent on activation of the inflammasome (Schroder and Tschopp, 2010). Within the inflammasome complex, the cleavage of pro-IL-1β and pro-IL-18 is mostly performed by caspase-1 with help of caspase-11 (Rathinam et al., 2012). Nevertheless, there is a fraction of cytokine maturation that is not performed by the caspase-1/-11 pathway (Mayer-Barber et al., 2010; Mcelvania Tekippe et al., 2010; Abdalla et al., 2012) but most likely by caspase-8 (Gringhuis et al., 2012). The NLR (nucleotide-binding and oligomerization domain, leucine-rich-repeat-containing) proteins such as NLRP3 and NLRC4 are one family of cytosolic receptors, which upon ligand binding mediate inflammasome activation. In the case of Mtb, most studies using *ex vivo* analysis of macrophages and dendritic cells identify NLRP3 as the sole NLR capable of inducing inflammasome activation (Carlsson et al., 2010; Mayer-Barber et al., 2010; Mcelvania Tekippe et al., 2010; Mishra et al., 2010; Wong and Jacobs, 2011; Abdalla et al., 2012; Dorhoi et al., 2012). Absent in Melanoma 2 (AIM2) is the best studied member of the HIN-200 family of DNA binding proteins, which is involved in the surveillance of the cytosol for double-stranded DNA (dsDNA) (Burckstummer et al., 2009; Fernandes-Alnemri et al., 2009; Hornung et al., 2009). Despite an apparent failure of Mtb to activate the AIM2-inflammasome *ex vivo, Aim2*^−/−^ mice are more susceptible to Mtb infections demonstrating a role for the AIM2 inflammasome *in vivo* and/or a putative inflammasome-independent function of AIM2 in host defense (Saiga et al., 2012). Here we will review and discuss the recent literature on the molecular mechanisms of the interaction of Mtb with NLRP3 and AIM2 inflammasomes and their connection with type I IFN signaling.

## Interaction of Mtb with host cell NLRP3-inflammasome

Infection of macrophages or dendritic cells deficient in NLRP3 with Mtb does not induce secretion of IL-1β or IL-18 (Carlsson et al., 2010; Mayer-Barber et al., 2010; Mcelvania Tekippe et al., 2010; Mishra et al., 2010; Wong and Jacobs, 2011; Abdalla et al., 2012; Dorhoi et al., 2012). The dogma for NLRs has been that they bind to a pathogen and/or danger associated molecular pattern (P/DAMP) in the host cell cytosol, which triggers their activation and subsequent formation of active inflammasomes. Nevertheless, the nature of such a ligand for NLRP3 has remained elusive. Recent publications suggest that there may not be a P/DAMP directly binding to NLRP3. Instead, cytosolic viral and bacterial RNA is sensed by DHX33, which then binds to and activates NLRP3 (Mitoma et al., 2013). Bacterial toxins, phagocytosis and other cellular insults result in potassium depletion in the cytosol, which serves as an activator of NLRP3 (Munoz-Planillo et al., 2013). Consistently, it was shown that potassium depletion is required for Mtb and non-tuberculous mycobacteria (NTM)-mediated inflammasome activation in macrophages (Kurenuma et al., 2009; Chen et al., 2012; Dorhoi et al., 2012; Lee et al., 2012, 2013). The protein tyrosine kinase Syk has been implicated in Mtb-mediated inflammasome activation (Wong and Jacobs, 2011), which is consistent with its previously identified role in activating the NLRP3-inflammasome after infection with the fungal pathogen *Candida albicans* (Gross et al., 2009). Another positive regulator is the thioredoxin-interacting protein that is activated by the loss of binding to thioredoxin after increase in cytosolic reactive oxygen species (ROS) and which subsequently binds to NLRP3 to activate it (Zhou et al., 2010). Nevertheless, this pathway is unlikely to be involved in Mtb-mediated NLRP3-inflammasome activation because inhibitors of ROS had no effect (Wong and Jacobs, 2011; Dorhoi et al., 2012). ROS-dependent and -independent pathways seem to converge at the mitochondrial membrane where the generation of the lipid cardiolipin can stimulate the NLRP3-inflammasome by binding to NLRP3 (Iyer et al., 2013). Finally, the guanylate binding protein 5 promotes NLRP3-inflammasome assembly and activation in response to bacterial infections as shown for *Listeria monocytogenes* and *Salmonella typhimurium* (Shenoy et al., 2012) but its role during Mtb infection has not been analyzed.

During the course of Mtb infections, the increase of IFN-γ in the lungs results in higher levels of nitric oxide (NO). Interestingly, NO has the capacity to negatively regulate the NLRP3-inflammasome and hence reduce IL-1β production (Mishra et al., 2013). The direct S-nitrosylation of NLRP3 accounts for the inhibitory effect of NO (Hernandez-Cuellar et al., 2012; Mishra et al., 2013). This aspect of NO activity is important to suppress tissue damage produced by continuous activation of innate immunity (Hernandez-Cuellar et al., 2012; Mishra et al., 2013). Another negative regulator of the NLRP3-inflammasome are omega-3 fatty acids which may prevent excessive inflammation and metabolic disorder (Yan et al., 2013). Furthermore, the LRRFIP2 binds to NLRP3 and also interacts with Flightless-1 to stimulate its binding to and subsequent inhibition of caspase-1 (Jin et al., 2013). The roles of these NLRP3 inflammasome regulatory pathways during Mtb infections have not been investigated.

## Interaction of Mtb with host cell AIM2-inflammasome

Intracellular bacterial pathogens such as *F. tularensis* and *L. monocytogenes* have been shown to induce the AIM2-inflammasome (Fernandes-Alnemri et al., 2010; Jones et al., 2010; Sauer et al., 2010; Tsuchiya et al., 2010). Interestingly, bacterial mutants of *F. tularensis* and *L. monocytogenes* in virulence factors that are important for bacterial access to host cell cytosol are deficient in activation of the inflammasome (Henry et al., 2007; Sauer et al., 2010). It is believed that during access to host cell cytosol some bacterial dsDNA also enters the cytosol where it will be recognized by AIM2 (Fernandes-Alnemri et al., 2010; Jones et al., 2010; Sauer et al., 2010). Indeed, AIM2 binds indiscriminately to cytosolic double-stranded DNA which could be of artificial (poly dA:dT), microbial or mammalian origin (Muruve et al., 2008; Rathinam et al., 2010).

**Figure 1:**
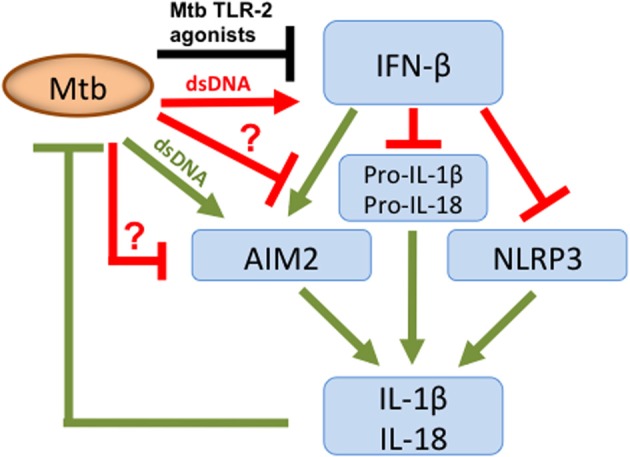
**Overview of Mtb host cell inflammasome interactions**. Mtb secretes dsDNA, which induces IFN-β production but also expresses TLR-2 agonists that inhibit. Nevertheless, overall there is an increase in IFN-β, which limits pro-IL-1β and pro-IL-18 transcription and has opposing effects on inflammasome activation. IFN-β inhibits the NLRP3-inflammasome but stimulates the AIM2-inflammasome. To limit AIM2-mediated IL-1β production, Mtb secretes putative effector(s) via its ESX-1 system, as indicated by “**?**.” They either directly or indirectly inhibit the AIM2-inflammasome resulting in diminished caspase-1 activation and subsequent reduction in IL-1β and IL-18 production. Green color denotes connections that are beneficial to the host; those in red are beneficial to the pathogen and in black those where this is unclear.

The type VII secretion system (ESX-1) of Mtb mediates translocation of Mtb extracellular DNA (eDNA) into the host cell cytosol where it can activate the DNA sensor IFI16/IFI204 which results in the initiation of a signaling cascade that ultimately leads to the production of IFN-β (Figure 1) (Stanley et al., 2007; Manzanillo et al., 2012). In the light of the indiscriminate binding of AIM2 to dsDNA it is surprising that the cytosolic Mtb eDNA does not induce activation of the AIM2 inflammasome. A clue on how to resolve this conundrum was offered by our recent finding that Mtb does not induce AIM2-inflammasome activation but other NTM, such as *M. smegmatis* (Msme), do (Shah et al., 2013). Msme has a functional ESX-1 system that is important for conjugal DNA transfer (Flint et al., 2004). Similar to the *L. monocytogenes* listeriolysin O mutant, the ESX-1 deficient Msme mutant showed a significant reduction in AIM2-inflammasome activation. Furthermore, Mtb could inhibit AIM2-inflammasome activation induced by infection with either Msme or the transfection of dsDNA (Shah et al., 2013). This inhibition by Mtb was dependent upon the presence of a functional ESX-1 system, since an ESX-1 deficient Mtb mutant failed to inhibit AIM2 activation (Shah et al., 2013). This suggests that the same secretion system that is responsible for introducing AIM2 ligands into the host cell cytosol may also transfer an effector that inhibits the AIM2 activation (Figure 1). The ESX-1 secretion system is also important for the escape of a fraction of intracellular Mtb from the phagosome into the cytosol (Van Der Wel et al., 2007; Houben et al., 2012; Simeone et al., 2012). The entry of Mtb into the cytosol could be another pathway for AIM2-inflammasome activation as it has been described for other bacteria that escape the phagosome (Brodsky and Monack, 2009). Nevertheless, Mtb escapes the phagosome at a very late stage (only after several days) during the infection (Houben et al., 2012; Simeone et al., 2012). In contrast, IL-1β production is usually analyzed within the first 24 h of infection and hence the current data in the literature on Mtb-inflammasome activation does not take the Mtb entry in the host cell cytosol into account. Analysis of these events during the late phase of the infection cycle would be very informative and may reveal Mtb-mediated AIM2-inflammasome activation.

The importance and probably also the limits of Mtb-mediated AIM2 inflammasome inhibition are highlighted by a study that demonstrates the increased susceptibility of *Aim2*^−/−^ mice and decreased levels of IL-1β and IL-18 in the lungs of these mice after Mtb infection (Saiga et al., 2012). The *ex vivo* analysis of macrophage infection in this study, demonstrating reduced IL-1β secretion in *Aim2*^−/−^ cells is in contradiction to many reports showing the sole dependence of BMDMs on NLRP3 for IL-1β production after Mtb infection. This may be due to the nature of macrophages used because in Saiga et al. thioglycollate-induced peritoneal macrophages were used as opposed to BMDMs in (Mayer-Barber et al., 2010; Mcelvania Tekippe et al., 2010; Mishra et al., 2010; Wong and Jacobs, 2011; Abdalla et al., 2012; Dorhoi et al., 2012). A more recent report that virulent *M. bovis* induce AIM2-inflammasome activation in a murine macrophage cell line further underscores the potential for AIM2-inflammasome in host defense against mycobacteria (Yang et al., 2013).

## The role of IFN-β in Mtb-inflammasome interactions

The significance of type I IFN signaling for activation of AIM2-inflammasome responses was first reported for *F. tularensis* and *L. monocytogenes* infected macrophages (Henry et al., 2007). Some reports demonstrate that the IFN-β acts at the level of increased transcription and translation of *Aim2* in order to increase AIM2-inflammasome activity (Jones et al., 2010; Tsuchiya et al., 2010). A conflicting report failed to detect changes in protein levels of AIM2 after infection with *F. tularensis* but confirmed the importance of this cytokine for AIM2-inflammasome activation (Fernandes-Alnemri et al., 2010). We reported that NTM induce secretion of up to 20fold higher levels of IFN-β when compared to Mtb infection (Shah et al., 2013). Furthermore, experiments using IFN-β neutralizing antibodies and *Ifnar1*^−/−^ deficient cells demonstrated a stimulatory role of IFN-β in the context of infections with NTMs (Shah et al., 2013). This is very comparable to the effect of IFN-β during *F. tularensis* or *L. monocytogenes* mediated inflammasome activation (Henry et al., 2007; Tsuchiya et al., 2010). Consequently, one could argue that minimizing IFN-β production will optimize the virulence of Mtb since there was an inverse correlation between IFN-β production and virulence. Nevertheless, the current paradigm is that an increase in type I IFN production leads to increased Mtb virulence (Manca et al., 2001, 2005; Stanley et al., 2007; Berry et al., 2010; Mayer-Barber et al., 2011; Manzanillo et al., 2012).

During the course of Mtb *ex vivo* infections of macrophages and dendritic cells no AIM2-inflammasome activation is detected. In this context, IFN-β has the opposite effect and suppresses activation of the NLRP3 inflammasome (Figure 1) (Mayer-Barber et al., 2011; Novikov et al., 2011). The mechanism of this inhibition involves the IFN-β mediated induction of IL-10 which suppresses IL-1β production (Mayer-Barber et al., 2011; Novikov et al., 2011). How then does Mtb inhibit the AIM2-inflammasome in the presence of IFN-β? The pre-infection of cells with Mtb inhibited IFN-β production induced by Msme in an ESX-1 dependent manner (Shah et al., 2013). This is in agreement with a previous report demonstrating that Mtb-induced TLR-2 signaling leads to depletion of IRAK1, which is required for TLR7/9-induced IFN-β production (Liu et al., 2012). These two reports show that Mtb is able to inhibit the production of IFN-β (Liu et al., 2012; Shah et al., 2013). Nevertheless, we demonstrated that the inhibition of IFN-β production is only a minor cause of the Mtb-mediated AIM2-inflammasome inhibition, since adding extracellular IFN-β did not completely overcome the inhibition (Shah et al., 2013). In addition, we showed that Mtb is capable of inhibiting autocrine IFN-β signaling (Shah et al., 2013). This observation is consistent with the previous report that Mtb inhibits IFN-α signaling, by reducing STAT1 phosphorylation and homodimer formation, since IFN-β and IFN-α signal through the same receptor (Prabhakar et al., 2003). This inhibition of IFN-β signaling may be linked to the inhibition of the AIM2 inflammasome but via a pathway that does not involve transcription or translational regulation of *Aim2*, since those were not affected by Mtb infection (Shah et al., 2013). Clearly, further investigation is needed to determine the molecular mechanism of Mtb mediated AIM2-inflammasome inhibition and its importance for bacterial virulence. In addition, the inhibition of IFN-β signaling could have the added benefit of reducing the production of IL-1β via the NLRP3 inflammasome. One example of a mechanism for such an inhibition could be the effect on caspase-11 activation, which is part of the IFNAR1 signaling pathway engaged by IFN-β. The activated caspase-11 synergizes with caspase-1 during NLRP3-inflammasome mediated maturation of pro-IL-1β (Rathinam et al., 2012). In conclusion, Mtb may have adapted to exploiting the pathogen-beneficial functions of IFN-β to exacerbate disease and inhibit the NLRP3-inflammasome by inhibiting potentially detrimental effects of IFN-β, which are the activation of the AIM2-inflammasome and autocrine IFN-β signaling (Figure 1).

Another point accentuated by our study (Shah et al., 2013) is that approaches comparing virulent Mtb with a broader spectrum of NTMs may help pointing toward inhibitory capacities of Mtb as it has done for the interaction of Mtb with host cell death (Keane et al., 2000; Velmurugan et al., 2007) and for host cell autophagy (Shin et al., 2010; Zullo and Lee, 2012). The long co-evolution of Mtb with humans and the selective pressure applied to human host defense to recognize and react to Mtb infections leads to the induction of innate immune responses. Nevertheless, Mtb, having been exposed to these responses, has adapted to counteract them, which is not the case for NTM. Consequently, comparing host responses quantitatively between virulent Mtb and NTM may offer clues to other Mtb-specific inhibitory mechanisms.

## Bacterial mediators of inflammasome inhibition

The inhibition of host cell inflammasome response as an immune evasion strategy has been described for viruses and bacterial pathogens (Taxman et al., 2010; Lamkanfi and Dixit, 2011; Vande Walle and Lamkanfi, 2011; Higa et al., 2013). The *Pseudomonas aeruginosa* secreted effectors ExoU and ExoS both mediate suppression of NLRC4-inflammasome dependent IL-1β production via unknown mechanisms (Sutterwala et al., 2007; Galle et al., 2008). The *Yersinia enterocolitica* proteins YopE and YopT both inhibit caspase-1 activation by targeting host cell Rac1 protein (Schotte et al., 2004). The *Y. pseudotuberculosis* YopK proteins bind to components of the bacterial type III secretion system to inhibit their recognition by host cell inflammasome (Brodsky et al., 2010). *Legionella* interferes with inflammasome activation by inhibiting transcription of the important inflammasome adapter gene *ASC* (Abdelaziz et al., 2011).

The Zn-metalloprotease, ZMP1, of Mtb has been implicated in inhibiting activation of the NLRP3-inflammasome (Master et al., 2008). Nevertheless, an independently generated *zmp1* Mtb deletion mutant failed to reproduce this phenotype (Wong and Jacobs, 2011). One reason for this difference may be that most experiments in the study by *Master et al*. were performed using a *zmp1* mutant in the background of the vaccine strain *M. bovis* BCG which lacks functional ESX-1 system. Another difference between the studies was the type of host cell being murine cells vs. human THP-1 cell line. The Mtb Rv3364c protein can bind to and inhibit host cell protease, cathepsin G (Danelishvili et al., 2011). This inhibition leads to a reduction in caspase-1 activity and pyroptosis of host cells (Danelishvili et al., 2011). The impact of the inhibition of caspase-1 on IL-1β production has not been addressed in that study. Cathepsin G has also been implicated in inflammasome independent processing of pro-IL-1β to mature IL-1β (Hazuda et al., 1990). Hence there are two potential pathways for the Rv3364c proteins to limit IL-1β production after Mtb infection. Our recent results suggest that an ESX-1-dependent secreted effector of Mtb is mediating the inhibition of host cell AIM2-inflammasome (Shah et al., 2013). In addition, other Mtb proteins may limit the NLRP3-inflammasome activation via inhibition of IFN-β signaling (Rathinam et al., 2012). In conclusion, there is a growing list of important human bacterial pathogens that have developed strategies to manipulate the host cell inflammasome, hence lending indirectly support to the argument that IL-1β production is important to host defense. The molecular mechanisms and especially the bacterial effectors involved in Mtb's interaction with host cell inflammasome are poorly defined and merit further investigations.

## Conclusions

The importance of IL-1β for host immune defense against Mtb infections is established (Mayer-Barber et al., 2010; Mcelvania Tekippe et al., 2010; Mayer-Barber et al., 2011). It is also clear that *in vivo* IL-1β levels are not affected by the absence of NLRP3 or ASC during Mtb infections, which highlights the importance of inflammasome-independent pathways (Mayer-Barber et al., 2010; Mcelvania Tekippe et al., 2010; Walter et al., 2010). Consistently, *Nlrp3*^−/−^ mice are not more susceptible and *Asc*^−/−^ mice are only slightly more susceptible to Mtb infections (Mayer-Barber et al., 2010; Mcelvania Tekippe et al., 2010; Dorhoi et al., 2012). Here we discussed recent reports demonstrating that Mtb can inhibit NLRP3-inflammasome activity via an IFN-β pathway and also either directly or indirectly inhibits the AIM2-inflammasome. In the light of these findings it is compelling to hypothesize that Mtb may actually be proficient at inhibiting other types of inflammasomes. Following this line of thought it may be that the *Nlrp3*^−/−^ and *Asc*^−/−^ mice have none to only minor phenotypes respectively, because Mtb is already so efficient at inhibiting their activity that even completely taking away these components of the host defense does not make much of a difference. Instead, host immune defense has evolved to develop inflammasome-independent pathways for IL-1β production to combat the highly adapted human pathogen Mtb. Serine proteases such as proteinase-3, elastase and cathepsin-G of neutrophils have been described to mature pro-IL-1β independently of inflammasome activation (Netea et al., 2010). It will be interesting to investigate if any of the two major cell populations responsible for IL-1β production in the lungs during Mtb infection use this mechanism to mature pro-IL-1β (Mayer-Barber et al., 2011).

### Conflict of interest statement

The authors declare that the research was conducted in the absence of any commercial or financial relationships that could be construed as a potential conflict of interest.
